# Serum surfactant protein D in COVID-19 is elevated and correlated with disease severity

**DOI:** 10.1186/s12879-021-06447-3

**Published:** 2021-08-03

**Authors:** Ming Tong, Ying Xiong, Chen Zhu, Hong Xu, Qing Zheng, Yu Jiang, Lianhong Zou, Xiaolin Xiao, Fang Chen, Xiquan Yan, Changping Hu, Yimin Zhu

**Affiliations:** 1 Department of Infectious Diseases, Hunan Provincial People’s Hospital (The First-affiliated Hospital of Hunan Normal University), Changsha, Hunan China; 2 Institute of Emergency Medicine, Hunan Provincial Key Laboratory of Emergency and Critical Care Metabonomics, Hunan Provincial People’s Hospital (The First-affiliated Hospital of Hunan Normal University), Changsha, Hunan China; 3 School of Life Sciences, Hunan Normal University, Changsha, Hunan China; 4 The Fourth People’s Hospital of Yiyang, Yiyang, Hunan China; 5 Department of Pediatrics, Yiyang Central Hospital, Yiyang, Hunan China; 6 Department of Geriatrics, Hunan Provincial People’s Hospital (The First-affiliated Hospital of Hunan Normal University), Changsha, Hunan China; 7 Department of Pharmacology, Xiangya School of Pharmaceutical Sciences, Central South University, Changsha, Hunan China

**Keywords:** Surfactant protein D, Coronavirus disease 2019, Disease severity stratification, Length of stay, Recovery phase

## Abstract

**Background:**

The serum surfactant protein D (SP-D) level is suggested to be a useful biomarker for acute lung injuries and acute respiratory distress syndrome. Whether the serum SP-D level could identify the severity of coronavirus disease 2019 (COVID-19) in the early stage has not been elucidated.

**Methods:**

We performed an observational study on 39 laboratory-confirmed COVID-19 patients from The Fourth People’s Hospital of Yiyang, Hunan, China. Receiver operating characteristic (ROC) curve analysis, correlation analysis, and multivariate logistic regression model analysis were performed.

**Results:**

In the acute phase, the serum levels of SP-D were elevated significantly in severe COVID-19 patients than in mild cases (mean value ± standard deviation (SD), 449.7 ± 125.8 vs 245.9 ± 90.0 ng/mL, *P*<0.001), while the serum levels of SP-D in the recovery period were decreased dramatically than that in the acute phase (mean value ± SD, 129.5 ± 51.7 vs 292.9 ± 130.7 ng/ml, *P*<0.001), and so were for the stratified patients. The chest CT imaging scores were considerably higher in the severe group compared with those in the mild group (median value, 10.0 vs 9.0, *P* = 0.011), while markedly lower in the recovery period than those in the acute phase (median value, 2.0 vs 9.0, *P*<0.001), and so were for the stratified patients. ROC curve analysis revealed that areas under the curve of lymphocyte counts (LYM), C-reaction protein (CRP), erythrocyte sedimentation rate (ESR), interleukin-6 (IL-6), and SP-D for severe COVID-19 were 0.719, 0.833, 0.817, 0.837, and 0.922, respectively. Correlation analysis showed that the SP-D levels were negatively correlated with LYM (r = − 0.320, *P* = 0.047), while positively correlated with CRP (r = 0.658, *P*<0.001), IL-6 (r = 0.471, *P* = 0.002), the duration of nucleic acid of throat swab turning negative (r = 0.668, *P*<0.001), chest CT imaging score on admission (r = 0.695, *P*<0.001) and length of stay (r = 0.420, *P* = 0.008). Multivariate logistic regression model analysis showed that age (*P* = 0.041, *OR* = 1.093) and SP-D (*P* = 0.008, *OR* = 1.018) were risk factors for severe COVID-19.

**Conclusions:**

Elevated serum SP-D level was a potential biomarker for the severity of COVID-19; this may be useful in identifying patients whose condition worsens at an early stage.

## Background

Since December 2019, a newly identified illness termed coronavirus disease 2019 (COVID-19) has spread rapidly through China and the rest of the world, and then severe acute respiratory syndrome coronavirus 2 (SARS-CoV-2) was identified as the pathogen [1]. As of July 1st, 2021, more than 182 million cases and 3.9 million deaths have been spotted worldwide [2].

Severe COVID-19 has been described as an immune dysregulated systemic infection, characterized by activation of T-helper-1 cell responses and elevating secretion of T-helper-2 cytokines that suppress inflammation, and cytokine storm is associated with disease severity [3]. The infection causes acute respiratory infection symptoms, from fever, cough, breathing difficulties, to acute respiratory distress syndrome (ARDS), and even leads to multi-organ failure or death.

As a glycoprotein mainly produced by alveolar type II cells, surfactant protein D (SP-D) is a member of the collectin family, involved in innate host defenses against microorganisms, and can also modulate adaptive immune responses. Meanwhile, SP-D is suggested to be a useful biomarker for acute lung injuries and ARDS [4] and plays a protective role in various causes of acute lung injury [5–7]. During the course of acute lung injury, increased serum SP-D levels are associated with lung functions [8]. However, whether the serum level of SP-D could identify the severity of COVID-19 in the early stage has not been elucidated.

In this study, we examined 39 laboratory-confirmed COVID-19 cases from The Fourth Hospital of Yiyang, Hunan China, to investigate whether the serum level of SP-D could stratify the disease severity at an early stage.

## Methodology

### Study design

An observational study including 39 confirmed COVID-19 adult patients from February 1, 2020, to March 10, 2020, was conducted in The Fourth People’s Hospital of Yiyang, Hunan, China. Laboratory-confirmed COVID-19 patients were defined as positive for real-time reverse-transcriptase polymerase-chain-reaction (RT-PCR) assay for throat-swab specimens. In our setting, patients had no pregnancy, and no history of autoimmune disorder, haematological disorder, malignant tumor, chronic obstructive pulmonary disease, or long-term treatment with warfarin, aspirin, or statins. The stratification of COVID-19 severity is according to the guidelines published by the National Health Council of China [9]. Among the patients, thirty were stratified into mild pneumonia, while nine were severe cases. As described in our previous study [10], all the participants received inhaled interferon α-2b and oral lopinavir-ritonavir as antiviral therapies. In the cohort, all patients survived during the observation period.

We extracted clinical characteristics and laboratory results from medical records. Blood routine tests, biochemical tests, quantifications of serum SP-D, plasma C-reaction protein (CRP), interleukin-6 (IL-6), lactic acid, D-dimer, erythrocyte sedimentation rate (ESR), and chest CT scanning were performed at admission.

Serum levels of SP-D and chest CT scanning were reexamined during the recovery period, i.e., the time that the patients who were afebrile for at least 72 h, significantly improved in lung lesions on chest CT, relieved from respiratory symptoms, and repeatedly negative in throat-swab specimens at least 24-h intervals.

Ethical approval for the study (No. 2020–10) was obtained from the ethics committee of Hunan Provincial People’s Hospital by the Code of Ethics of the World Medical Association Declaration of Helsinki. All study participants provided written informed consent.

### Clinical data collection

Demographic data, underlying diseases, laboratory findings, chest CT imaging, and treatment measures were extracted from medical records. All data were analyzed and triple-checked by three physicians.

### CT imaging scoring

The chest CT images were analyzed by two radiologists with extensive experience in thoracic radiology and scored using an existed system described before [11]. The scoring details were described in Table 1.

**Table 1:** CT imaging performance and corresponding score system

Number	CT Imaging performance	Score
1	unbilateral patchy shadows or ground glass opacity	5
2	bilateral patchy shadows or ground glass opacity	7
3	diffuse changes for (1) or (2)	2
4	unbilateral solid shadow or strip shadow	2
5	bilateral solid shadow or strip shadow	4
6	unbilateral pleural effusion	2
7	bilateral pleural effusion	4
8	increased or enlarged mediastinal lymph nodes	1

### Sample collection and processing

AT 6 a.m. the next day after admission, blood sampling was collected for each patient by standard venipuncture in a fasting state and repeated on the day of discharge. White blood corpuscle counts (WBC), lymphocyte counts (LYM), and the serum levels of CRP, ESR, IL-6, lactic acid, and D-dimer were measured by conventional laboratory methods. Serum for SP-D detection was isolated by centrifugation for 15 min at 1500×g and frozen at − 80 °C until thawed once and analyzed using commercially available Human SP-D enzyme-linked immunosorbent assays (ELISA) kits (Boster Biological Technology Co. Ltd., Wuhan, China). The intra-assay and inter-assay coefficients of variation were 2.6 and 3.1%, respectively. The sensitivity was calculated to be 0.02 ng/mL.

### Data analysis

Categorical variables were expressed as the number [proportions] and compared using χ2 analysis. Continuous variables were expressed as mean and standard deviation (SD), median and interquartile range (IQR) values. Independent group *t*-tests was used to compare means for continuous variables that were normally distributed, while the Mann-Whitney *U* test was used for continuous variables that were not normally distributed. Intra-group comparisons during follow-up were performed by paired-samples *t*-test or *Wilcoxon* test. All statistical analyses were performed using SPSS 19.0 (SPSS Inc., Chicago, IL, USA). Correlation analyses were performed by Pearson’s correlation coefficient. Multivariate logistic regression was conducted for risk analyses of disease severity. A two-sided *P*-value of less than 0.05 was considered statistically significant.

## Results

The median age for COVID-19 patients was 49 years (IQR: 31–56), and 20 (51.3%) of them were men. There were no considerable differences in gender, current smokers, diabetes history, cardiovascular disease history, and WBC between the mild and severe group, while significant differences were observed in age, LYM, and serum levels of CRP, ESR, IL-6, D-dimer, and lactic acid (Table 2). Compared with the mild pneumonia group, the severe group had significantly higher serum SP-D levels at admission (449.7 ± 125.8 vs 245.9 ± 90.0 ng/ml, *P*<0.001, Table 2). The median duration of the nucleic acid of throat swab turning negative (DNA-N) after diagnosis for the mild and severe groups was 8.5 and 14.0 days, respectively (*P* = 0.004), and the medium duration of the length of stay (LOS) was 10.6 and 16.6 days (*P*<0.001), respectively (Table 2).

**Table 2:** Demographics and laboratory findings of COVID-19 patients

	Overall	Mild	Severe	*P*-value
Gender(M/F), n/n	20/19	16/14	4/5	0.720
Age (years)^a^	49 (31–56)	49 (25–55)	54 (47–75)	0.030
Current Smokers, n (%)	4 (10)	2 (7)	2 (22)	0.223
Diabetes History, n (%)	4 (10)	2 (7)	2 (22)	0.223
CVD History, n (%)	3 (8)	1 (3)	2 (22)	0.127
WBC(×10^9^/L)^a^	6.27 (4.64–7.82)	5.70 (4.34–7.66)	6.42 (5.47–8.98)	0.243
Lymphcytes(×10^9^/L)^a^	1.16 (0.84–1.68)	1.31 (1.02–1.78)	1.04 (0.59–1.16)	0.049
CRP (mg/L)^a^	3.50 (0.50–16.50)	0.59 (0.50–5.06)	38.9 (17.3–66.6)	0.000
ESR (mm/h)^a^	22.0 (12.0–39.5)	20.2 (10.6–29.4)	41.7 (27.0–76.8)	0.004
IL-6(pg/mL)^a^	21.6 (12.0–30.8)	11.19 (7.45–15.54)	18.71 (17.20–32.52)	0.002
D-dimer (mg/L)^a^	0.43 (0.19–0.88)	0.35 (0.15–0.52)	4.49 (1.29–7.00)	0.000
Lactic Acid (mmol/L)^a^	0.88 (0.68–1.54)	0.80 (0.68–1.17)	1.95 (1.15–2.25)	0.004
SP-D (ng/mL)	292.9 ± 130.7	245.9 ± 90.0	449.7 ± 125.8	0.000
CT imaging score^a^	9.0 (5.0–11.0)	9.0 (5.0–9.5)	10.0 (9.0–15.0)	0.011
Length of stay (days)	12.0 ± 4.3	10.6 ± 3.5	16.6 ± 3.5	0.000
DNA-N (days)^a^	10.0 (6.0–14.0)	8.5 (5.0–11.8)	14.0 (11.0–17.0)	0.004

Values are mean ± standard deviation (SD) if not otherwise stated, or number [proportions]. ^a^Median (25, 75 percentile), *P*-values refer to comparison between mild and severe COVID-19 patients

Abbreviations: *CVD* Cardiovascular Disease; *WBC* white blood corpuscle; *CRP* C-reaction protein; *ESR* erythrocyte sedimentation rate; *IL-6* interleukin-6; *SP-D* surfactant protein D; *DNA-N* duration of nucleic acid of throat swab turning negative

Detectable abnormalities, with typical manifestations of bilateral ground-glass opacity and sub-segmental consolidation, were observed in chest CT images for all patients on admission. Not surprisingly, the imaging scores presented dramatically higher in the severe group compared with those in the mild group. (median value, 10.0 vs 9.0, *P* = 0.011, Table 2).

**Fig. 1:**
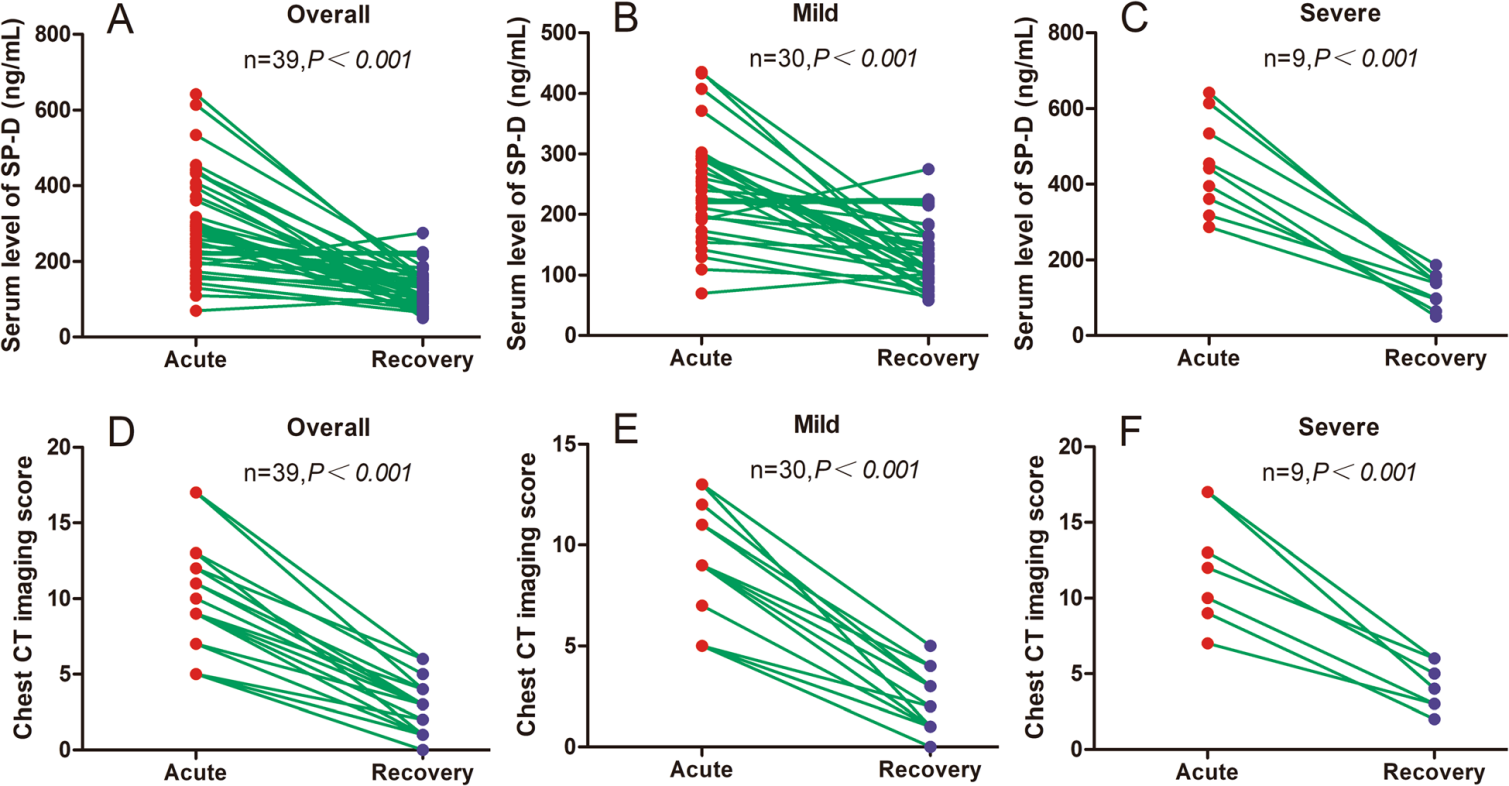
Serum surfactant protein D (SP-D) levels and chest computed tomography imaging scores were compared in the acute and recovery phase for overall, mild and severe patients

While compared with the acute phase, the serum SP-D levels were decreased significantly in the recovery period for overall patients (292.9 ± 130.7 vs 129.5 ± 51.7 ng/ml, *P*<0.001; Fig. 1A), in line with the mild group (194.5 ± 66.1 vs 120.0 ± 50.0 ng/ml, *P*<0.001; Fig. 1B), and the severe group (449.7 ± 125.8 vs 119.8 ± 45.2 ng/ml, *P*<0.001; Fig. 1C). Chest CT imaging scores in the recovery period for overall patients was markedly decreased than those in the acute phase (median value, 2.0 vs 9.0, *P*<0.001; Fig. 1D), consistent with the mild group (median value, 2.0 vs 5.0, *P*<0.001; Fig. 1E), and the severe group (median value, 1.0 vs 3.0, *P*<0.001; Fig. 1F).

**Fig. 2:**
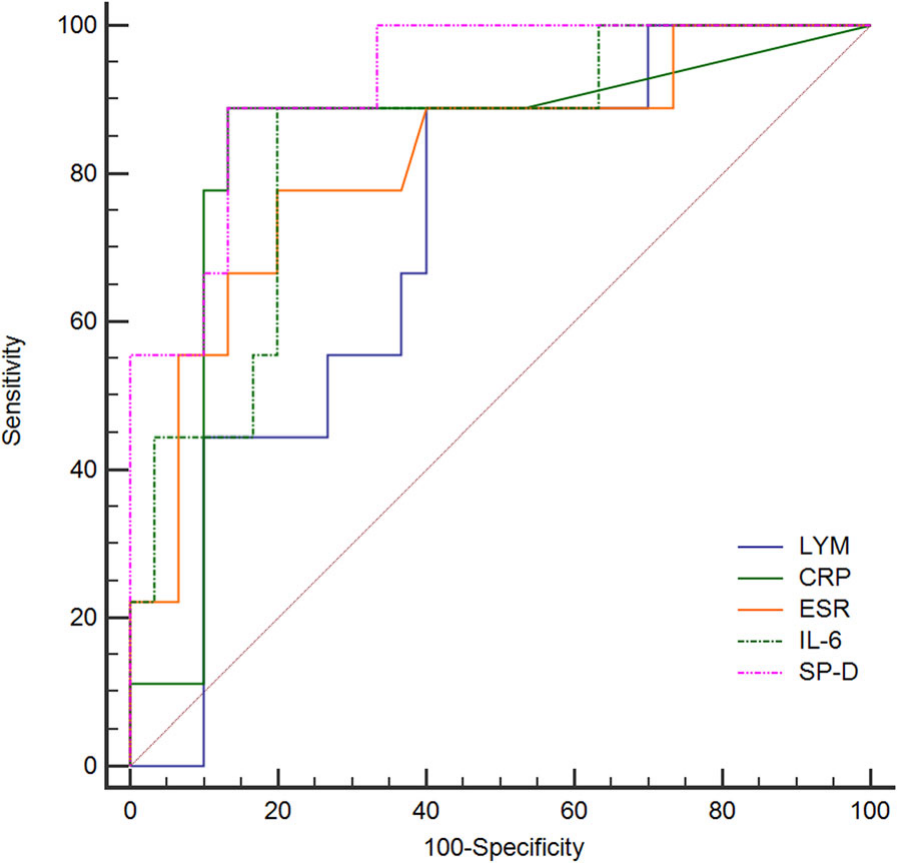
Receiver operating characteristic curve of lymphocyte counts (LYM), C-reaction protein (CRP), erythrocyte sedimentation rate (ESR), interleukin-6 (IL-6), and serum surfactant protein D (SP-D)

To test the potency of inflammatory markers in distinguishing severe from mild cases, receiver operating characteristic (ROC) curve analysis was performed. The area under the curve (AUC) of lymphocyte counts was 0.719 (95% confidence interval (CI) 0.546–0.851, *P* = 0.049), and the optimum cutoff was 1.18 × 10^9^/L, (sensitivity 60.0%, specificity 88.9%). The AUC of CRP was 0.833 (95%CI 0.664–1.000, *P* = 0.003), and the optimum cutoff was 9.05 (sensitivity 88.9%, specificity 86.7%). The AUC of ESR was 0.817 (95%CI 0.651–0.983, *P* = 0.004), and the optimum cutoff was 31.7 (sensitivity 77.8%, specificity 80.0%). The AUC of IL-6 was 0.837 (95%CI 0.690–0.984, *P* = 0.002), and the optimum cutoff was 16.4 (sensitivity 88.9%, specificity 80.0%). The AUC of SP-D was 0.922 (95%CI 0.833–1.000, *P*<0.001), and the optimum cutoff was 309.7 (sensitivity 88.9%, specificity 86.7%) (Fig. 2).

**Fig. 3:**
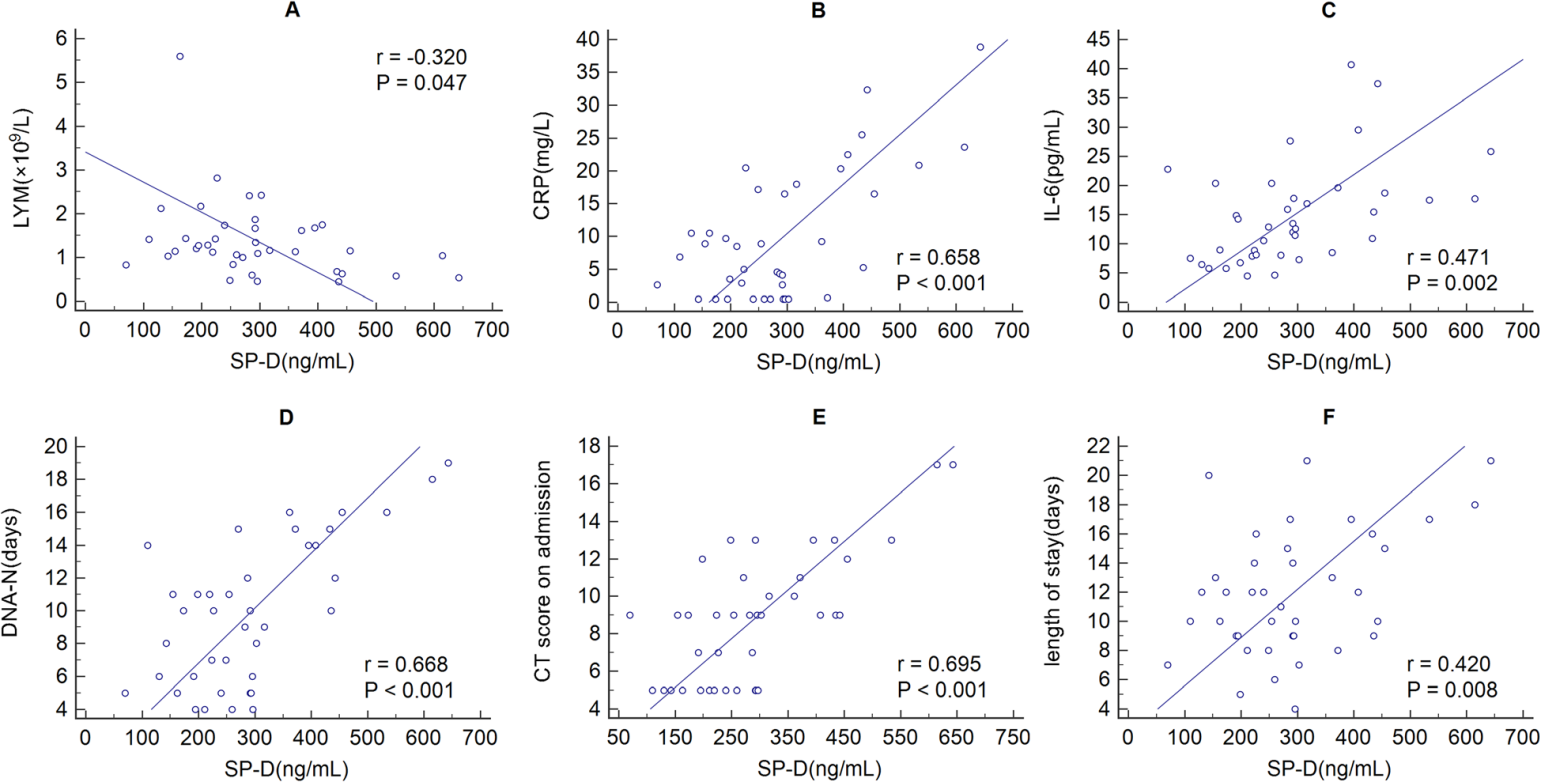
Correlation analysis between SP-D and LYM, CRP, ESR, IL-6, DNA-N, chest CT imaging score, and length of stay

There was a significant negative correlation between SP-D levels and lymphocyte counts (r = − 0.320, *P* = 0.047), while positively correlations between SP-D with CRP (r = 0.658, *P*<0.001), IL-6 (r = 0.471, *P* = 0.002), DNA-N (r = 0.668, *P*<0.001), CT imaging score on admission (r = 0.695, *P*<0.001) and length of stay (r = 0.420, *P* = 0.008) were observed (Fig. 3A-F). No dramatic correlation between SP-D and WBC counts, ESR, lactic acid, and D-dimer were observed (Data not shown).

Age, LYM, CRP, IL-6, ESR, and SP-D were used to establish the multivariate logistic regression model. The results revealed that age (*P* = 0.041, *OR* = 1.093, 95%CI 1.004 ~ 1.191) and SP-D (*P* = 0.008, *OR* = 1.018, 95%CI 1.005 ~ 1.032) were risk factors for severe illness (Table 3).

**Table 3:** Results of multivariate logistic regression analysis

Variable	B	SE	Wald *χ*^2^	*P*	OR	OR 95% CI
age	0.089	0.044	4.170	0.041	1.093	1.004 ~ 1.191
SP-D	0.018	0.007	7.068	0.008	1.018	1.005 ~ 1.032
Constant	−12.005	3.756	10.214	0.001	0.000	

## Discussion

In the present study, we found for the first time that in the acute phase, the serum SP-D level was elevated in patients with severe COVID-19 pneumonia compared with mild patients, suggesting that the serum SP-D level was closely related to the disease severity and would be an early potential biomarker of disease severity for stratifying the COVID-19 patients on admission. We also found that the serum SP-D levels were significantly decreased in the recovery period compared with that on admission for the overall COVID-19 patients, or the stratified COVID-19 patients.

In the cohort, severe cases were associated with elder age, which is in line with the previous report [12], suggesting that COVID-19 is more vulnerable for those aged due to weaker immune functions [13]. In terms of laboratory findings, lymphopenia and elevation of inflammatory biomarkers (CRP, ESR, IL-6), and higher levels of D-dimer and lactic acid were associated with severe patients. Little has been observed on hyperlactatemia in severe COVID-19 patients. Infection-associated hyperlactatemia is due to tissue hypoxia and anaerobic glycolysis, and the increased aerobic glycolysis is secondary to the activation of the stress response [14]. Due to the systemic hypoxemia, coagulopathy, physical and psychological stress in COVID-19 patients [15], we assume that hyperlactatemia is prevalent in severe COVID-19 patients and may contribute to the disease progression.

Moreover, severe cases had longer hospitalization and higher CT imaging scores. Since CT imaging scores differ in intensive care unit (ICU) patients and non-ICU patients, it is suggested that the CT imaging scores associate with the severity of COVID-19 pneumonia [3]. In our study, the elevation of serum SP-D levels was positively associated with longer hospitalization duration, an indicator of slow recovery, and higher CT imaging scores, suggesting that the serum SP-D level may serve as a biomarker of disease severity for stratifying the COVID-19 patients at an early stage, the results of ROC analysis and multivariate logistic regression analysis also confirmed this.

SP-D is mainly secreted into the alveoli and is considered to be a candidate marker for alveolar integrity [16], thus elevated systemic SP-D levels are considered to be due to the leakage of pulmonary proteins into the circulation [17], reflecting the permeable abnormality of alveolar-capillary membrane, possibly due to loss of its structural and functional integrity [18]. Meanwhile, SP-D participates in innate immune responses, and the essential function of SP-D is to clear pathogens by agglutination, opsonization [19], and to modulate the function of macrophages and dendritic cells [20, 21]. Further, it has been reported that SP-D clears SARS-CoV through direct interaction with the viral spike glycoprotein [22], and SARS-CoV-2 attaches to angiotensin-converting enzyme 2 (ACE2) receptors by spike glycoproteins to enter the airway and lung epithelia [23]. Due to the similarity of spike glycoprotein domain between SARS-CoV-2 and SARS-CoV [23], it is speculated that SP-D may play a protective role in the binding of SARS-CoV-2 to ACE2. However, severe COVID-19 patients exhibit sustained systemic hyper-inflammation state and cytokine storms, characterized by lymphopenia and the elevation of TNF-alpha, IL-1, and IL-6 [24]. During the systemic hyper-inflammation state and cytokine inflammation, IL-6 induces the release of SP-D into the systemic circulation [25]. In our cohort, the serum levels of IL-6 and SP-D were elevated dramatically in severe patients, suggesting that SP-D, together with IL-6, may participate in the complex immune dysregulation process and contribute to the pathogenesis of severe COVID-19. Taken together, whether SP-D plays a protective or detrimental role in the pathogenesis of COVID-19 still warrants further studies.

Furthermore, the synthesis and secretion of SP-D upsurge during acute lung injury and continue to increase during persistent inflammation [26], which implies that SP-D may be a potential biomarker for chronic pulmonary complications in COVID-19. Although the serum level of SP-D was markedly decreased in the recovery period in our setting patients, whether SARS-Cov-2 infection causes long-term lung sequelae remains to be observed.

Limitations to this study should be noted. Firstly, the small sample size with 39 cases has the risk of false-negative associations. The sample size was estimated by G*Power 3.1.9.7 to be 45 cases with an actual power of 0.95. Considering that the COVID-19 pandemic resulted in global healthcare crises and post-acute COVID-19 syndromes, as well as the valuable follow-up data during the recovery phase, we believe that our study with 39 cases is still worth sharing. Secondly, we did not measure the levels of SP-D in bronchoalveolar samples, and as such, the relationship between the serum level of SP-D and lung injury is still uncertain, and a more extensive study is needed. Thirdly, our study focused on the stratification value of the serum SP-D level in the acute phase of severe patients and mild cases, as well as its changes in the recovery phase; therefore, no comparison with a healthy control group is one of the limitations.

## Conclusion

The present study demonstrated that the serum SP-D level was significantly increased in severe COVID-19 patients in the acute phase and decreased in the recovery period, which was related to the disease severity. The present results warrant further studies consolidating that the serum SP-D levels may assess the progression of COVID-19-associated lung injury, providing a means for monitoring disease severity at an early stage.

## Data Availability

The datasets used and/or analysed during the current study are available from the corresponding author on reasonable request.

## Abbreviations

ACE2: angiotensin-converting enzyme 2
ARDS: acute respiratory distress syndrome
CI: confidence interval
COVID-19: coronavirus disease 2019
CRP: C-reaction protein
CT: computed tomography
CVD: Cardiovascular Disease
DNA-N: duration of nucleic acid of throat swab turning negative
ELISA: enzyme-linked immunosorbent assays
ESR: erythrocyte sedimentation rate
IL-2: interleukin-2
IL-6: interleukin-6
LYM: lymphocyte counts
LOS: length of stay
ICU: intensive care unit
IQR: interquartile range
ROC: Receiver operating characteristic
RT-PCR: reverse-transcriptase polymerase-chain-reaction
SARS-CoV-2: severe acute respiratory syndrome coronavirus 2
SP-D: surfactant protein D
WBC: white blood corpuscle
